# Induced spawning with gamete release from body ruptures during reproduction of *Xenoturbella bocki*

**DOI:** 10.1038/s42003-023-04549-z

**Published:** 2023-02-17

**Authors:** Hiroaki Nakano, Ako Nakano, Akiteru Maeno, Michael C. Thorndyke

**Affiliations:** 1 Shimoda Marine Research Center, University of Tsukuba, 5-10-1, Shimoda, Shizuoka 415-0025 Japan; 2 Kristineberg Marine Research Station, University of Gothenburg, Kristineberg 566, Fiskebäckskil, 45178 Sweden; 3 Cell Architecture Laboratory, National Institute of Genetics, Yata 1111, Mishima, Shizuoka 411-8540 Japan

**Keywords:** Zoology, Taxonomy

## Abstract

*Xenoturbella* is a marine invertebrate with a simple body plan, with recent phylogenomic studies suggesting that it forms the phylum Xenacoelomorpha together with the acoelomorphs. The phylogenetic position of the phylum is still under debate, whether it is an early branching bilaterian or a sister group to the Ambulacraria. Phylogenetic traits often appear during development, and larva resembling the cnidarian planula has been reported for *Xenoturbella*. However, subsequent developmental studies on *Xenoturbella* have been scarce. This is mainly due to the difficulties in collecting and keeping adult animals, resulting in the lack of data on the reproduction of the animal, such as the breeding season and the spawning pattern. Here we report on the reproduction of *X. bocki* and confirm that its breeding season is winter. Spawning induction resulted in gametes being released from body ruptures and not the mouth. No evidence supported the animal as a simultaneous hermaphrodite.

## Abstract (web summary)

The marine invertebrate *Xenoturbella bocki* breeds in the winter and the induction of spawning leads to gamete release through body ruptures, not the mouth.

## Introduction

*Xenoturbella* is a marine benthic animal possessing a very simple body plan^1,2^. It lacks a through gut, gonads, coelomic cavities, and brain, with its nervous system being organized as a diffuse basiepidermal nerve net. Due to the simple body plan, various phylogenetic positions have been suggested for this animal, such as basal metazoans, basal bilaterians, flatworms, acoels, hemichordates, holothurians, bryozoans, and molluscs (reviewed in refs. ^1,2^). Recent studies, including molecular phylogenomic analyses, agree that *Xenoturbella* forms a phylum, Xenacoelomorpha, together with the acoels and nermatodermatids^3–8^. Within the phylum, *Xenoturbella* is regarded to be the early branching clade, with acoels and nermatodermatids being sister groups^3–7^. However, the phylogenetic position of the phylum is still under debate, whether it is an early branching bilaterian^3,4^ or a sister group to the Ambulacraria (echinoderms + hemichordates)^5–7^.

Another important remaining problem of *Xenoturbella* is how the animal obtained such a simple body plan. In connection with its undecided phylogenetic position, it is still not known whether it retains the simple body plan of the last common ancestor of bilaterians, or if it was secondarily simplified from the last common ancestor of xenambulacrarians (Xenacoelomorpha + Ambulacraria). The evolutionary history of a species is often reflected in its development, and this may also be the case for *Xenoturbella*. Its larva showed little similarities with the dipleurula-type larva known from ambulacrarians and resembles the planula larva of cnidarians^9^, seemingly supporting the early branching bilaterian theory for Xenacoelomorpha. Transcriptomic analyses during development and microstructural studies on larvae shall be useful in further verifying this hypothesis, but there have been no ensuing studies on xenoturbellid development, except for a report on its cleavage pattern^1^. The main reason for this lack of research is the difficulties in collecting mature adult specimens and keeping them alive in laboratories, with most species living in sea bottoms deeper than 200 m^10,11^. Even for *X. bocki*, a species that inhabits exceptionally shallow waters at around 100 m, information on its reproduction and breeding season is scarce^12^. In the original description of the species, it was reported to be hermaphrodites, based on a section with mature sperm and immature oocytes^13^. However, no figures were presented showing individuals possessing both types of mature gametes, and whether it is a simultaneous hermaphrodite remains uninvestigated. The breeding season was reported as winter based on a few specimens^13^. The egg is suggested to be released from the mouth, but no figures were shown to support this^13^. Internal fertilization was suggested in the original description^13^, but sperm morphology revealed by recent studies suggested external fertilization^14^. Internal fertilization was also implied from the presence of embryos and larvae within the adult body^15,16^, but it has been argued that these embryos and larvae may be ingested prey. To sum up, many of the knowledge on *Xenoturbella* reproduction is based on details provided in the original description of the species^13^, but some of the statements lack supporting data and have not been since tested by other researchers.

We have made observations on *X. bocki* reproduction, which indicate that the breeding season is winter. We also report artificially induced spawning of *Xenoturbella* using potassium chloride, with the gametes being released mainly from a newly formed hole usually at the posterior terminal end of the animal. We did not obtain evidence supporting internal fertilization or simultaneous hermaphroditism. We hope these findings will be valuable for future studies on *Xenoturbella* reproduction and development.

## Results

### Breeding season

**Fig. 1:**
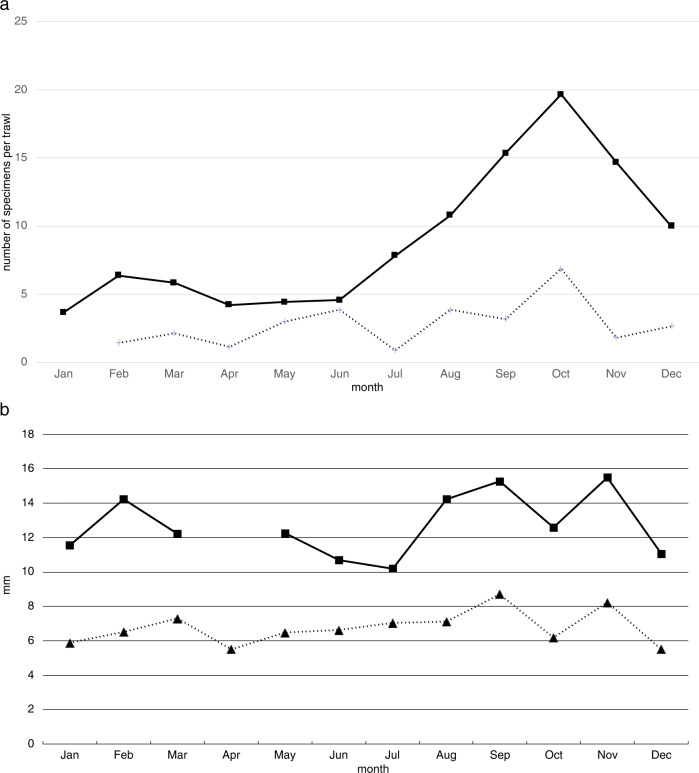
Monthly collections of *Xenoturbella bocki*. **a** Number of collected specimens per trawl. **b** The average length of collected animals. In the graphs, solid lines show specimens collected off Skar in 2006 (*n* = 590), and dotted lines show specimens collected off Lysekil in 2005 (*n* = 197), both locations situated within Gullmarsfjord.

**Fig. 2:**
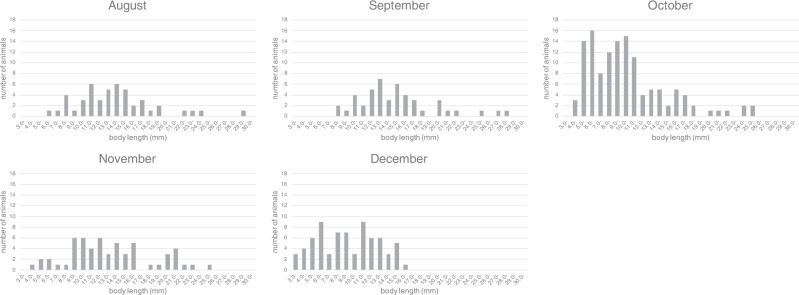
Size distribution of *Xenoturbella bocki* collected from August to December within Gullmarsfjord from 2005 to 2006. August *n* = 47, September *n* = 46, October *n* = 127, November *n* = 57, December *n* = 72.

Monthly collections of *Xenoturbella bocki* revealed that the number of collected specimens was highest in October in two different locations within Gullmarsfjord, Sweden (Fig. 1a). On the other hand, the average length of collected animals was low in October (Fig. 1b). Looking at the size distribution of animals from the same population collected during August to December, large numbers of small specimens was collected in October when compared with other months (Fig. 2).

**Fig. 3:**
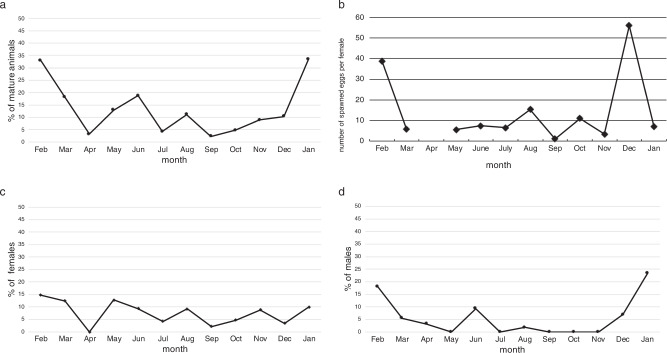
Spawning induction of *Xenoturbella bocki* performed from 2006 to 2007. **a** Percentage of animals that spawned eggs or sperm in which spawning was induced (*n* = 556). **b** Average number of spawned eggs per female (*n* = 47). **c** Percentage of animals that spawned eggs in which spawning was induced (*n* = 556). **d** Percentage of animals that spawned sperm in which spawning was induced (*n* = 556).

During the period of February 2006 to February 2007, spawning induction was performed on the collected animals every month (see next section for details on spawning). The percentage of animals with mature gametes had a clear peak in winter, with over 30% of the animals being mature (Fig. 3a). The average number of eggs per female was also highest in winter (Fig. 3b). Some mature females were found every month except for April, but mature males were rarely found from July to November (Fig. 3c, d). Based on these observations, the breeding season of *X. bocki* was confirmed as winter, in accordance with the statement made in the original description of the species^13^.

### Spawning

**Fig. 4:**
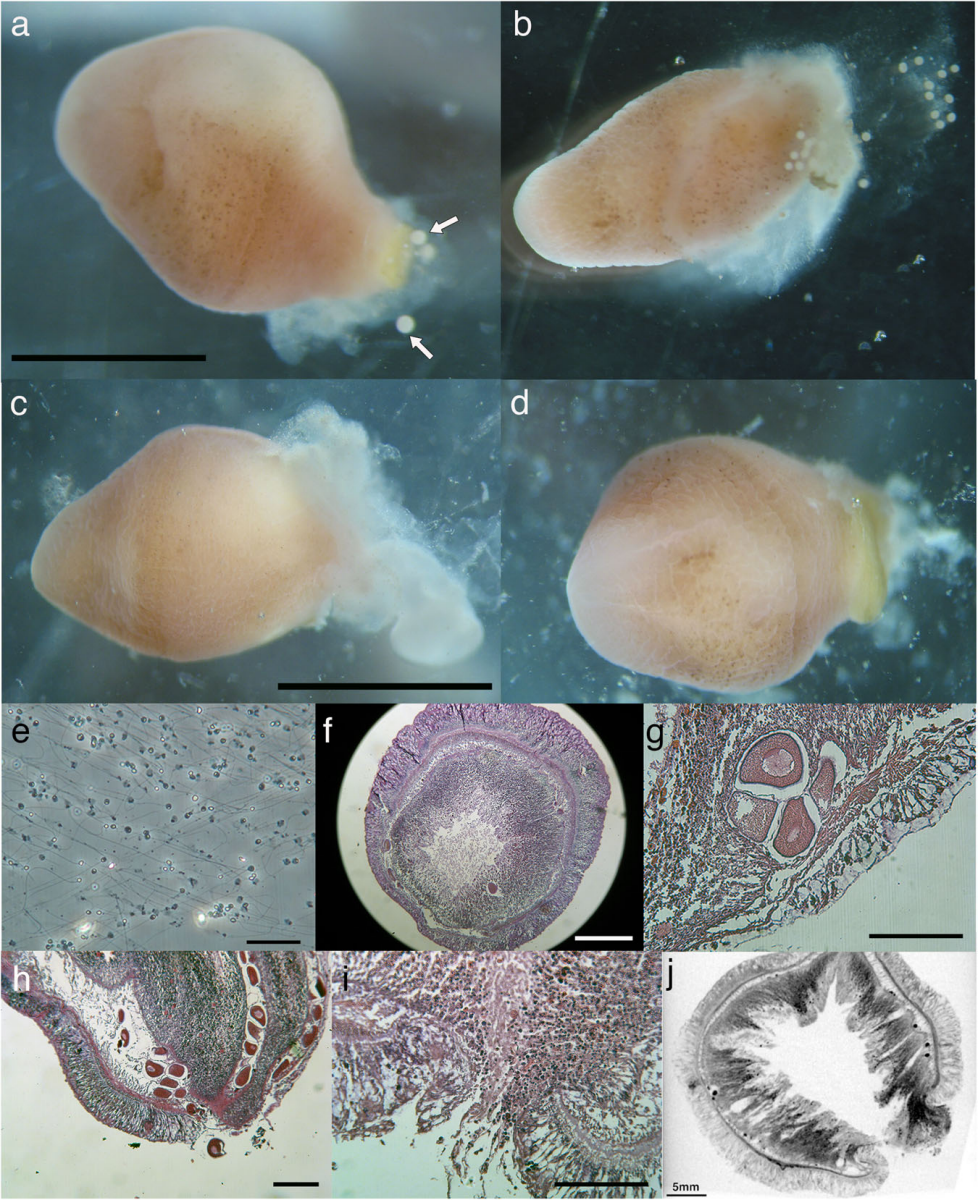
Spawning of *Xenoturbella bocki*. Female (**a**, **b**) and male animals (**c**, **d**) spawn gametes together with white mucus from their posterior ends. Dorsal view with the anterior to the left. White arrows: eggs. **e** Sperm present in the mucus released by the male specimen is shown in (**d**). **f** Section of a female specimen. The epidermis is thin, and the subepidermal membrane complex (SMC) are scarce at the bottom left of the picture. **g** Posterior end of a female specimen where the epidermis is thin and SMC are scarce. **h** A rupture formed at the posterior end of a female. **i** A close-up of a rupture formed at the posterior end of a female. **j** Coronal microCT section of an animal with a rupture that connects to the gut cavity. Scale bars: **a**, **c**, **j**: 5 mm; **e**: 10 μm; **f**: 500 μm; **g**–**i**: 200 μm.

Applying potassium chloride solution (KCl) is the traditional method to induce spawning in sea urchins^17^, and it has been shown to be effective in other marine animals such as sea cucumbers, bivalves, and limpets^18–20^. When KCl was applied to the collected xenoturbellids, gametes were released from some of the animals. In females, eggs were released from new openings in the body together with white to translucent mucus (Fig. 4a, b). In males, white mucus was released from the new openings (Fig. 4c, d). The presence of sperm in the mucus was confirmed by observations by light microscopy (Fig. 4e). The new openings were formed mainly at the posterior terminal end of the animal (Table 1). Sections revealed that adult *Xenoturbella* has parts of the body where the epidermis is thin and the subepidermal membrane complex (SMC) are scarce or even missing, especially at the posterior end (Fig. 4f, g). The hole had no epithelial tissues or back lining muscles or nerves, suggesting that it is a rupture within the body wall and not a preexisting structure such as a gonopore (Fig. 4h, i). The hole sometimes penetrated through the gut wall and connected to the gut cavity (Fig. 4j), but often it only connected the space filled with pseudoparenchymal cells between the epidermis and the gut (Fig. 4h). The hole was not observed externally after a few days, supposedly being closed using muscles.

**Table 1:** Site of rupture after spawning induction in *Xenoturbella bocki*.

Site	Number of animals
Anterior	8
Posterior	194
Posteroventral	33
Ventral	26
Dorsal	7
Either of the lateral sides	24
Multiple ruptures present	33

### Gametes within the adult body

By performing morphological studies on xenoturbellids collected during the breeding season, we investigated the position of eggs and sperm within the adult body.

**Fig. 5:**
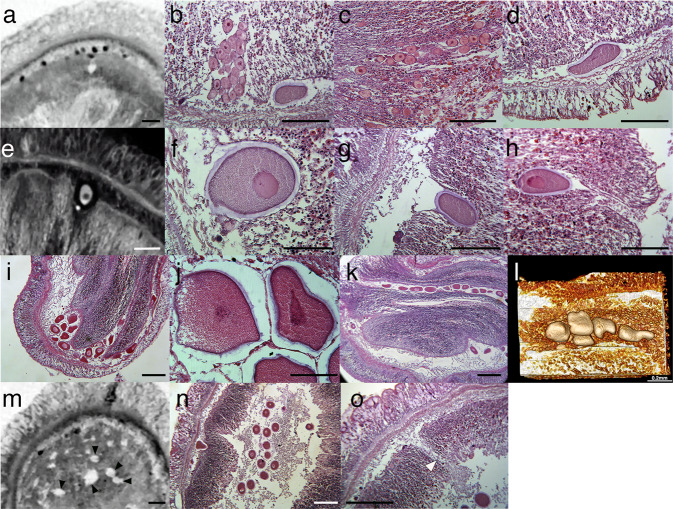
Oocytes and eggs inside *Xenoturbella bocki*. **a** MicroCT section showing immature oocytes as black spots present between the epidermis and the gut. **b** A group of oocytes present in a female. **c** Oocytes lined up in a near single row. **d** Mature egg found between the epidermis and the gut. **e** MicroCT section showing an oocyte in a pocket sinking within the gut. **f** Mature egg in a pocket sinking within the gut. **g** Mature egg that has almost sunk within the gastrodermal cells. **h** Mature egg that has sunk within the gastrodermal cells. The pocket is connected by a thin tunnel to the space between the epidermis and the gut. **i** A group of eggs present in a female. **j** The eggs in a group is divided by a thin layer into individual compartments. **k** Eggs lined up in a near single row in a female. **l** Volume rendering image from microCT scans showing five eggs within a female. The eggs are not in a complete single row. **m** MicroCT section showing empty pockets (black arrowheads) present in the gut. **n** Eggs present in the gut cavity. **o** A tunnel (white arrowhead) connecting the space between the epidermis and the gut and the gut cavity. Scale bars: **a**–**e**, **g**–**i**, **k**–**o**: 200 μm; **f**, **j**: 100 μm.

In females, oocytes and eggs within the body were pressed between different organs and were not spherical or disc-shaped (Fig. 5). Therefore, it was impossible to measure the exact size of the oocytes and eggs from histology and microCT observations and hence the approximate diameter of the oocytes and eggs are indicated in the following sentences.

Immature oocytes approximately 30-80 μm in diameter were found mostly at the outer thin layer of the gut or between the epidermis and the gut (Fig. 5a). The oocytes were often found in groups, sometimes exceeding 20 in number (Fig. 5b). Occasionally, the oocytes were observed to be lined up in a near single row at the outer thin layer of the gut (Fig. 5c).

Larger oocytes roughly 100–150 μm in diameter and mature eggs about 200 μm in diameter were mostly found in three sites within the female body. First, they were present in the space between the epidermis and the gut, with no apparent attachment to either tissue (Fig. 5d). Second, they were found in pockets sinking within the digestive organ (Fig. 5e–h). In cases where multiple large oocytes are present inside a single pocket (Fig. 5i), the pocket was divided into compartments by thin layers so that each compartment contains a single oocyte (Fig. 5j). Large oocytes were sometimes found to be lined up in pockets in a near single row in some specimens (Fig. 5k, l). Empty pockets, possibly those that previously held eggs, were present in some specimens (Fig. 5m). Third, larger oocytes and mature eggs were found within the gut cavity (Fig. 5n), apparently unaffected by the digestive function of the organ. A tunnel with no epithelial tissues or back lining structures connected the space between the epidermis and the gut and the gut cavity in some specimens (Fig. 5o). The pockets and tunnels maybe the same structure as canals previously reported in the gastrodermis of females for *Xenoturbella*^15^.

**Fig. 6:**
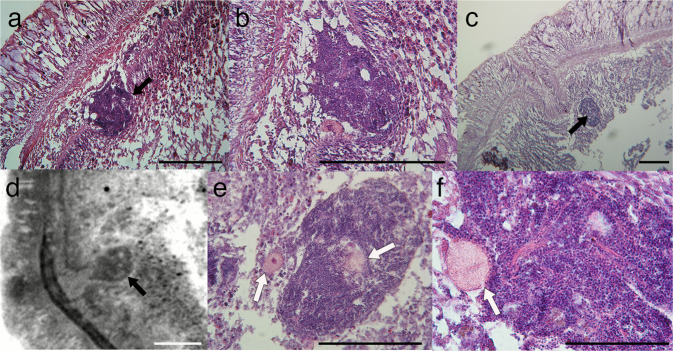
Sperm inside *Xenoturbella bocki*. **a**, **b** Sperm cluster located on the surface of the gut. **c**, **d** Sperm cluster situated within the gastrodermal cells. **e**, **f** Sperm clusters with oocytes within or near the clusters. Black arrows: sperm clusters, white arrows: oocytes. Scale bars: **a**–**d**: 200 μm; **e**, **f**: 100 μm.

In males, sperm was found in clusters on the outer layer of the gut (Fig. 6a, b) or within the gut cells (Fig. 6c, d). The sperm clusters were unstructured, and the epithelial tissue surrounding them were not evident. There seemed to be no specific arrangement of the sperm within the clusters. The clusters sometimes contained immature eggs within or near them (Fig. 6e, f). Sections revealed that all the animals, including the ones that spawned mature sperm and those that did not possess mature gametes, had oocytes within their bodies. The oocytes were found mostly at the outer thin layer of the gut but were also often present between the epidermis and the gut, within the cells of the digestive organ, and inside the digestive cavity.

No fertilized eggs, embryos, or larvae were found inside adult *Xenoturbella* either by spawning induction or sectioning, throughout the year.

## Discussion

Large numbers of small animals, about 4–6 mm in length, were collected in October in comparison with other months (Fig. 2). Since the newly settled *X. bocki* specimens are about 255 μm in length^9^ and the average length of the animals can increase about 2 mm in 1 month (Fig. 2), it is likely that individuals born in winter reach the body size that can be collected using a 1 mm sieve by October. The small animals showed rapid and synchronized growth during autumn, with the average length of the collected animals increasing about 2 mm between September and October (Fig. 2), and the percentage of small individuals being low in November and December (Fig. 2). Therefore, it is possible that the small animals collected in October join the reproducing population in winter, implying that individuals reach maturity in 1 year in *X. bocki*. Since the average length does not change drastically after the breeding season, the animals probably do not die after spawning. The observation that the animals in which spawning was induced survived the treatment and spawning also supports this view. Hence, the lifespan of the animal likely exceeds one year. Although data were not available for growth rates after maturity, the presence of large animals exceeding 3 cm from the area suggests that *X. bocki* may live for a few years. It would be of interest to investigate the growth rate and lifespan of *X. monstrosa* inhabiting the eastern Pacific Ocean, whose body length exceeds 20 cm^10^.

We report spawning of *Xenoturbella bocki*, occurring from a new opening in the body wall (Fig. 4). The hole had no back lining structure such as muscles or nerves (Fig. 4h, i), suggesting they are ruptures formed for spawning. Of the animals with at least one hole, 59.7% had a hole at the posterior end (Table 1). Egg laying from body ruptures is not rare in animals, known from such animals as gastrotrichs, rotifers, and Platyhelminthes, as well as acoels and nemertodermatids, belonging to the same phylum as xenoturbellids (reviewed in ref. ^21^). These observations suggest that spawning from body ruptures may be normal for *Xenoturbella*. However, as spawning was induced using potassium chloride in this study, this behavior may not portray the natural spawning behavior. Although heavily damaged individuals and animals with apparent holes were excluded before the experiments, it may be possible that the ruptures were concealed injuries obtained during collections that got enhanced by the chemical spawning induction.

Another possibility is that the gametes are spawned from the mouth. In the original description of *X. bocki*, the author suspected spawning to occur from the mouth, since it is the only opening normally present^13^. Although eggs were present in the space between the epidermis and the gut (Fig. 5), a tunnel connected the space and the gut cavity in some specimens (Fig. 5o), and we regard it as possible to spawn all eggs from the mouth without making new ruptures. More observations are needed to determine where the gametes are released from during natural spawning behavior in *Xenoturbella*.

No animals spawned fertilized eggs, embryos, or larvae during this study. Although sectioning revealed the presence of orthonectids in some of the animals^22^, no fertilized eggs, embryos, or larvae were found inside the body. Both methods were performed throughout the year. Furthermore, other species with similar primitive sperm morphology^14^ have external fertilization. Moreover, the larva of *X. bocki* swims in a similar pattern as larvae of other marine invertebrate animals with development outside the adult body^9^. These facts suggest that *Xenoturbella bocki* has external fertilization and development outside the adult body. We regard that this mode of reproduction is ancestral to xenacoelomorphs, and that internal fertilization accompanied by derived sperm was acquired in acoelmorphs after xenoturbellids branched off.

*Xenoturbella* is regarded to live in low densities, as dredging large areas often results in only few specimens. External fertilization is an inefficient way to reproduce such an animal. However, an abundance of *X. bocki* has been suggested to be as high as 111 individuals/m^3^ in February^23^, and it is possible that the animals aggregate during the breeding season and spawn simultaneously. Whether this behavior is based on pheromones or on taxis to a certain environmental stimulus will be a focus of future research.

We found 47 animals with mature eggs and 34 with sperm (Fig. 3), implying that the sex ratio is approximately 1:1 during breeding season. *X. bocki* have been reported to be hermaphrodites^13^, and we found immature oocytes in individuals with mature sperm in the present study (Fig. 6). However, we did not find any simultaneous hermaphroditic individuals possessing both mature sperm and mature eggs. It may be possible that *X. bocki* change sex during the breeding season, but none of the animals that were treated with potassium chloride one month after the original spawning induction had reversed its sex. Another possibility is that *X. bocki* possess both male and female immature gametes and mature only a single type of gametes in a breeding season. Gonochorism has been reported for congeneric species *X. profunda*^10^, and further studies are necessary to uncover the evolution and diversity of sexual systems in *Xenoturbella*.

**Fig. 7:**
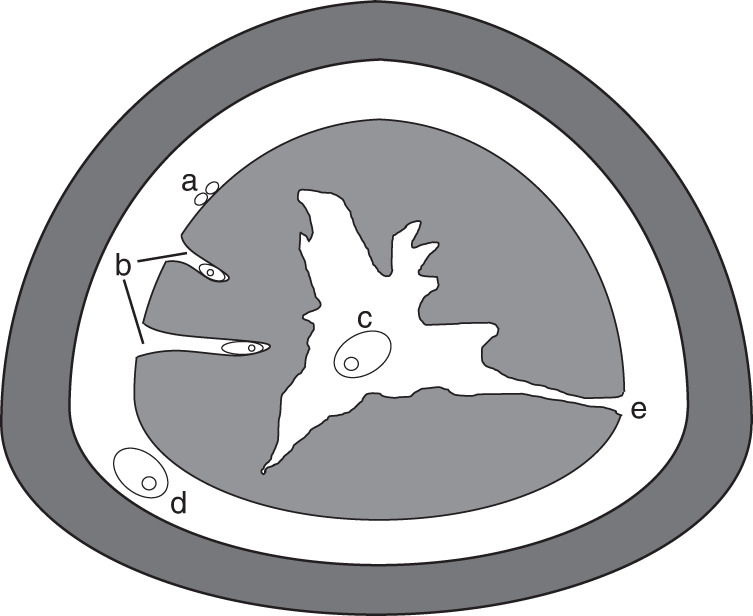
Gametogenesis of *Xenoturbella*. Schematic drawing showing a transverse section of *Xenoturbella*. **a** Germ cells are present on the surface of the gut. **b** Gametes sink into pockets within the gut as they mature. Mature gametes are present in the gut cavity (**c**) or between the epidermis and the gut (**d**). **e** Tunnel connecting the two spaces are present in some specimens.

From our results, we theorize that gametogenesis in *Xenoturbella* proceeds as follows (Fig. 7). Germ cells are mainly present on the surface layer of the gut. At the onset of the breeding season, maturation of gametes begins at the site. As maturation proceeds, the gametes sink into pockets within the gastrodermal cells. In females, this presumably occurs when oocytes reach about 100–150 μm in diameter. The gametes are released inside the gut cavity after maturation (when eggs are about 200 μm in diameter in females), but since there are tunnels connecting the space between the epidermis and the gut and the gut cavity, gametes may move from the latter space to the former and back. At spawning, the gametes are discharged from a rupture in the body wall, mainly situated at the posterior end. Although not observed in our study, it is possible that gametes present in the gut cavity are spawned from the mouth.

We have reported here the reproductive features of *Xenoturbella* and hope the information from this study shall become the basis for observing the complete development of the animal. Developmental studies on *Xenoturbella*, such as transcriptomic analyses and microstructural studies during the developmental stages, shall help clarify its phylogenetic position and shall uncover important clues for elucidating bilaterian or deuterostome evolution.

## Methods

### Sampling

Adult *Xenoturbella bocki* were collected using the research vessel and Warren’s dredge of the Kristineberg Marine Research Station, University of Gothenburg as reported in Nakano 2015^1^. Mud acquired from the sea bottom is sieved and the animals are found from the sieved material. Collections were performed at two locations within Gullmarsfjord, Sweden: off Skar at 80–120 m depth and off Lysekil at 50–90 m depth. The exact sampling site was changed each time to allow the sea bottom to recover from the damage that may be caused by the dredge collections. Animals for this study was mainly collected from 2005 to 2007, but additional collections were performed from 2015 to 2017. Collected xenoturbellids were kept in containers with mud and running natural seawater at Kristineberg Marine Research Station.

### Spawning induction

The collected animals were placed in cooled 0.25 M potassium chloride (KCl) in filtered seawater for 20–40 min. After the treatment, the animals were transferred into seawater and kept at 4–6 °C. Pictures were taken with a Canon S40 color camera adapted to a Leica stereomicroscope MZFLIII microscope and analyzed with Adobe Photoshop. The presence of sperm in the released mucus was investigated by placing the mucus on a slide glass, putting a coverslip on the mucus, and observing it with a Leica light microscope type DBRBE. The treated animals survived for months after the induction without any apparent negative effects.

### Histological sections

Animals were fixed in 4% paraformaldehyde in filtered seawater overnight at 4 °C. They were then dehydrated in a series of ethanol and embedded in paraffin. Sections (6 μm) were placed on glass slides, dewaxed, and stained with hematoxylin-eosin. Photographs of the sections were taken under a Leica light microscope type DBRBE and analyzed with Adobe Photoshop.

### MicroCT

MicroCT was performed according to the protocols for *Xenoturbella* in ref. ^24^. Samples were fixed, stored, and stained under the conditions described in Supplementary Table 1. The stained samples were scanned using an X-ray microCT system (ScanXmate-E090S105; Comscan Techno) at a tube voltage peak of 60 kV and a tube current of 110 μA. During scanning, samples were rotated 360 degrees in steps of 0.14 to 0.18 degrees, generating 2000–2500 projection images. Images were reconstructed using the software provided with the X-ray microCT system (coneCTexpress; Comscan Techno). 2D and 3D tomographic images were obtained using the OsiriX (www.osirix-viewer.com) software program.

### Reporting summary

Further information on research design is available in the Nature Portfolio Reporting Summary linked to this article.

## Supplementary information

Supplementary Information

Reporting Summary

## Data Availability

All data were available from the corresponding author on reasonable request.
