# Supplementary material for: Induced spawning with gamete release from body ruptures during reproduction of Xenoturbella bocki
Source: Commun Biol. 2023 Feb 17;6:172. doi: 10.1038/s42003-023-04549-z (PMC9938242; doi:10.1038/s42003-023-04549-z)
Supplement: Supplementary file 1 — Supplementary Information [file 42003_2023_4549_MOESM1_ESM.pdf]

Supplementary Table 1 : Sample preparation and scanning conditions for microCT observations

	Fixation	Staining		MicroCT Scanning									Data analysis	
**specimen**	**Fixation and storage**	**solution**	**Time**	**Scanning medium**	**Scanned parts**	**Scanned method**	**Voltage and current**	**Frame average**	**Frame rate**	**Rotation steps**	**Number of projections**	**Voxel size (μm)***	**Dataset size (GB)****	**Figure**
060320 #7 F	4% PFA 70% ethanol	20%Lugol	1day	0.5% agarose	whole body	Normal	60kV 110μA	4	8 fps	0.18°	2000	4.2	0.97	Fig. 4j, 6e
060320 #8 F	4% PFA 70% ethanol	20%Lugol	2days	0.5% agarose	whole body	Normal	60kV 110μA	4	8 fps	0.18°	2000	5.3	0.90	Fig. 6a, 6m
					oocyte	Pinpoint	60kV 110μA	4	8 fps	0.14°	2500	2.9	0.97	Fig. 6l
060224 #13 M	2.5% glutaraldehyde 0.1 M sodium-cacodylate	20%Lugol	1day	DW	whole body	Multi-step	60kV 110μA	2	8 fps	0.18°	2000	2.5	1.43	Fig. 7d

* : Isotropic voxel resolution

** : Total size (gigabyte) of 8bit TIFF format image files
