# Supplementary material for: Induced spawning with gamete release from body ruptures during reproduction of Xenoturbella bocki
Source: Commun Biol. 2023 Feb 17;6:172. doi: 10.1038/s42003-023-04549-z (PMC9938242; doi:10.1038/s42003-023-04549-z)
Supplement: Supplementary file 2 — Reporting Summary [file 42003_2023_4549_MOESM2_ESM.pdf]

nature portfolio

Corresponding author(s): Hiroaki Nakano

Last updated by author(s): Jan 26, 2023

Reporting Summary

Nature Portfolio wishes to improve the reproducibility of the work that we publish. This form provides structure for consistency and transparency in reporting. For further information on Nature Portfolio policies, see our Editorial Policies and the Editorial Policy Checklist.

Statistics

For all statistical analyses, confirm that the following items are present in the figure legend, table legend, main text, or Methods section.

n/a	Confirmed	
☐	☒	The exact sample size (*n*) for each experimental group/condition, given as a discrete number and unit of measurement
☒	☐	A statement on whether measurements were taken from distinct samples or whether the same sample was measured repeatedly
☒	☐	The statistical test(s) used AND whether they are one- or two-sided<br>*Only common tests should be described solely by name; describe more complex techniques in the Methods section.*
☒	☐	A description of all covariates tested
☒	☐	A description of any assumptions or corrections, such as tests of normality and adjustment for multiple comparisons
☒	☐	A full description of the statistical parameters including central tendency (e.g. means) or other basic estimates (e.g. regression coefficient) AND variation (e.g. standard deviation) or associated estimates of uncertainty (e.g. confidence intervals)
☒	☐	For null hypothesis testing, the test statistic (e.g. *F*, *t*, *r*) with confidence intervals, effect sizes, degrees of freedom and *P* value noted<br>*Give P values as exact values whenever suitable.*
☒	☐	For Bayesian analysis, information on the choice of priors and Markov chain Monte Carlo settings
☒	☐	For hierarchical and complex designs, identification of the appropriate level for tests and full reporting of outcomes
☒	☐	Estimates of effect sizes (e.g. Cohen's *d*, Pearson's *r*), indicating how they were calculated

Our web collection on statistics for biologists contains articles on many of the points above.

Software and code

Policy information about availability of computer code

Data collection	Raw data for microCT scans were collected using the built-in reconstruction software in the micro-CT system ScanXmate-E090S105, Comscantechno.
Data analysis	OsiriX DICOM Viewer (www.osirix-viewer.com), was used for analyzing microCT data. Data was analyzed and figures were created using Adobe Photoshop, Microsoft Excel, and Powerpoint.

For manuscripts utilizing custom algorithms or software that are central to the research but not yet described in published literature, software must be made available to editors and reviewers. We strongly encourage code deposition in a community repository (e.g. GitHub). See the Nature Portfolio guidelines for submitting code & software for further information.

Data

Policy information about availability of data

All manuscripts must include a data availability statement. This statement should provide the following information, where applicable:

- Accession codes, unique identifiers, or web links for publicly available datasets
- A description of any restrictions on data availability
- For clinical datasets or third party data, please ensure that the statement adheres to our policy

All data are available from the corresponding author on reasonable request.

Human research participants

Policy information about studies involving human research participants and Sex and Gender in Research.

Reporting on sex and gender	Not applicable.
Population characteristics	Not applicable.
Recruitment	Not applicable.
Ethics oversight	Not applicable.

Note that full information on the approval of the study protocol must also be provided in the manuscript.

Field-specific reporting

Please select the one below that is the best fit for your research. If you are not sure, read the appropriate sections before making your selection.

☐ Life sciences ☐ Behavioural & social sciences ☒ Ecological, evolutionary & environmental sciences

For a reference copy of the document with all sections, see nature.com/documents/nr-reporting-summary-flat.pdf

Ecological, evolutionary & environmental sciences study design

All studies must disclose on these points even when the disclosure is negative.

Study description	Studies on Xenoturbella reproduction using live and fixed specimens.
Research sample	Xenoturbella bocki
Sampling strategy	Xenoturbella bocki were collected regularly in Gullmarsfjord, Sweden, using a marine biological dredge.
Data collection	Authors H.N. and A.N. performed the collections and experiments on live animals and made observations on the sections. H.N. and A.M. performed microCT observations.
Timing and spatial scale	Animals were collected roughly once a month in Gullmarsfjord, Sweden mainly over a two year period.
Data exclusions	Not applicable.
Reproducibility	Collection were performed mainly over a 2 year period to check for the reproducibility on the breeding season. Other observations were performed on multiple specimens.
Randomization	Not applicable.
Blinding	Not applicable.

Did the study involve field work? ☒ Yes ☐ No

Field work, collection and transport

Field conditions	Xenoturbella bocki were collected regularly in Gullmarsfjord, Sweden,
Location	Xenoturbella bocki were collected in Gullmarsfjord, Sweden, from about 100 meters depth.
Access & import/export	Xenoturbella bocki were collected using research vessels Oscar von Sydow and Arne Tiselius of Kristineberg Marine Research Station, University of Gothenburg, Sweden. The animals were transported to the station by the vessels.
Disturbance	The sampling site was changed each time to allow the sea bottom to recover from the damage that may be caused by the dredge collections.

Reporting for specific materials, systems and methods

We require information from authors about some types of materials, experimental systems and methods used in many studies. Here, indicate whether each material, system or method listed is relevant to your study. If you are not sure if a list item applies to your research, read the appropriate section before selecting a response.

Materials & experimental systems

n/a	Involved in the study
☒	☐ Antibodies
☒	☐ Eukaryotic cell lines
☒	☐ Palaeontology and archaeology
☐	☒ Animals and other organisms
☒	☐ Clinical data
☒	☐ Dual use research of concern

Methods

n/a	Involved in the study
☒	☐ ChIP-seq
☒	☐ Flow cytometry
☒	☐ MRI-based neuroimaging

Animals and other research organisms

Policy information about studies involving animals; ARRIVE guidelines recommended for reporting animal research, and Sex and Gender in Research

Laboratory animals	This study did not involve laboratory animals.
Wild animals	This study did not involve wild animals (live vertebrates or higher invertebrates).
Reporting on sex	Not applicable.
Field-collected samples	This study did not involve animals (live vertebrates or higher invertebrates) collected from the field .
Ethics oversight	No ethical approval or guidance was required, as this study did not involve live vertebrates or higher invertebrates

Note that full information on the approval of the study protocol must also be provided in the manuscript.
